# Capturing Conformational Transitions of Fluorescently‐Coupled Polyelectrolyte Brushes with High Spatiotemporal Resolution

**DOI:** 10.1002/smll.202409323

**Published:** 2025-01-21

**Authors:** Jyoti Yadav, Ilka Hermes, Andreas Fery, Quinn A. Besford

**Affiliations:** ^1^ Leibniz‐Institut für Polymerforschung e. V Hohe Str. 6 01069 Dresden Germany

**Keywords:** conformation sensing, conformational changes, FRET, messenger materials, polyelectrolyte brushes

## Abstract

Polyelectrolyte brushes (PEBs) undergo conformational transitions due to changes in pH and/or ionic strength, which is leveraged as smart surfaces and on‐demand drug‐release systems. However, probing conformational transitions of functional PEBs has remained challenging due to low spatiotemporal resolution of characterization methods. Herein, fluorescently‐coupled PEBs are devised that give rise to Förster Resonance Energy Transfer (FRET) intrinsically coupled to conformational transitions of chains. Polyelectrolyte poly(2‐(dimethylamino)ethyl methacrylate) brushes are grown on silica surfaces via a grafting‐from approach, producing nanoscale brushes ≈60 nm in solvated height. The study chose to investigate pH as astimulus, at constant ionic strength, using pH‐insensitive fluorophores coupled within the brush (donors) and on‐chain ends (acceptors), leading to conformational FRET. The influence of pH on the FRET brushes is proved by ellipsometry and fluorescence spectroscopy. Importantly, using FRET meant chain conformation is spatially resolved with sub‐micrometer resolution by confocal laser scanning microscopy, where subtle changes in brush conformation are resolved in seconds. Unique mixing dynamics of different pH microdroplets on the brushes are identified as coalescence occurred, with reversible output, and a clear delay in brush responses to mixing liquids. The surfaces offer a new basis for probing conformational transitions of PEBs with high spatiotemporal resolution.

## Graphical abstract

Herein, polyelectrolyte brushes are devised with integrated FRET capabilities for revealing chain conformation. The systems provide a significant improvement in resolution for resolving pH‐induced changes in polyelectrolyte conformations, revealing new dynamics and memory effects of polyelectrolytes undergoing conformational transitions.

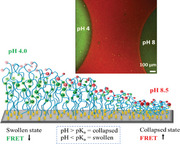

## 1 Introduction

Polyelectrolytes are the building blocks of numerous biological macromolecules, including DNA, proteins, and carbohydrates, and hence are crucial to the functioning of living organisms.^[^ ^1^ ^]^ Changes in the molecular conformation and interactions of polyelectrolyte segments are caused by pH, ionic strength, and temperature, which are responsible for a variety of biological functions to occur. This is due to their capability to dissociate into polyvalent macroions and counterions in an aqueous medium.

Polyelectrolytes can be surface‐grafted by conjugating the chain ends to a substrate with a relatively small grafting distance compared to the radii of gyration of the chains in solution, which are called polyelectrolyte brushes (PEBs). These multifunctional surfaces adapt to environmental changes and have attracted much interest in the last few years.^[^ ^2^, ^3^ ^]^ They can switch their conformation, brush height, charge distribution, degree of ionization, and wetting properties in the presence of an external trigger, such as pH, ionic strength, temperature, and counterions, to name a few.^[^ ^4^, ^5^, ^6^, ^7^, ^8^ ^]^ Specifically, PEBs undergo conformational transitions between a solvated (more hydrophilic) and collapsed (more hydrophobic) state as a function of stimuli, resulting from reversible microphase separation or self‐organization phenomena caused by ionic interactions, hydrogen bonding, and/or hydrophobic interactions.^[^ ^9^, ^10^ ^]^ This opens up new applications, such as transducers for label‐free sensors,^[^ ^11^ ^]^ and allows the construction of nanoscale actuators,^[^ ^12^ ^]^ such as those produced by local variations in stimuli. There remain some open questions regarding the mechanism of the brush response to variations in the solution stimuli, including changes in polymer conformation, degree of dissociation, and charge distribution through the brush.^[^ ^5^ ^]^ The use of PEBs for sensing stimuli fluctuations has largely been limited to methods that cannot readily spatially and/or temporally resolve the responses, and in some cases without damaging the system (invasively), such as “scratch” atomic force microscopy (AFM), along with spectroscopic ellipsometry, surface plasmon resonance (SPR), quartz crystal microbalance‐dissipation monitoring (QCM‐D), and water contact angle (WCA), as examples.^[^ ^13^ ^]^ To spatially resolve these responses, “messenger material” capabilities need to be integrated. These can involve amplification motifs that are plasmonic or fluorescence‐based, capable of analyzing nearby nanoscopic environments and converting this information into a signal that can further be transduced, or received, macroscopically by microscopy methods (i.e., spatially).^[^ ^14^ ^]^

Previous work by us focused on integrating these messenger material capabilities into poly(*N*‐isopropylacrylamide) (PNIPAM) brushes.^[^ ^15^ ^]^ This leveraged Förster Resonance Energy Transfer (FRET) chemistry in order to resolve conformational transitions. The basis of FRET is a non‐radiative transfer of energy from an excited donor fluorophore to an acceptor fluorophore when the fluorophores are sufficiently close (separations less than several nm), leading to a red‐shift in the fluorescence emission. This allows proximity changes between species to be resolved by careful analysis of the energy of the emitted fluorescence. However, these previous brush systems were intrinsically limited to PNIPAM with specific fluorophores, in diblock random copolymer architectures, synthesized by a grafting‐to approach, with minimal freedom to develop into other functional brush systems.^[^ ^15^ ^]^ We look to overcome these limitations by devising a new strategy for synthesizing functional PEBs that have FRET integrated to non‐invasively reveal details on polymer brush conformational transitions in real‐time with high spatial and temporal resolution.

Herein, we report on FRET‐integrated poly(2‐(dimethylamino)ethyl methacrylate) p(DMAEMA) brushes assembled using a surface‐initiated photoinduced electron transfer reversible addition‐fragmentation chain transfer polymerization (SI‐PET‐RAFT) approach. The weak polyelectrolyte p(DMAEMA) was specially chosen due to its exceptional sensitivity to subtle changes in solution pH,^[^ ^16^, ^17^ ^]^ which is our chosen stimulus to study PEB conformational transitions. The polymer chains were purposefully engineered with pH‐insensitive fluorophores to collect and transduce FRET responses from swelling/collapse transitions in chain conformation only. The FRET chemistry was integrated with donor fluorophores randomly in the bulk brush, whilst the acceptor fluorophores were specifically conjugated to chain ends. This allowed for the brush transitions as a function of stimuli to be spatially resolved by confocal laser scanning microscopy (CLSM), with high spatial (toward nanoscale) and temporal resolution (sub‐seconds). This is significantly greater than other methods for resolving PEB conformational changes, which can either not spatially resolve changes, or are limited to 10s of micrometers (e.g., ellipsometry), damaging the sample (AFM), and typically take 10s of minutes for stabilized signal and/or scanning.^[^ ^13^ ^]^ To the best of our knowledge, our FRET PEBs are the first systems for resolving PEB conformational transitions in such spatiotemporal resolution.

## 2 Results and Discussion

**Scheme 1:**
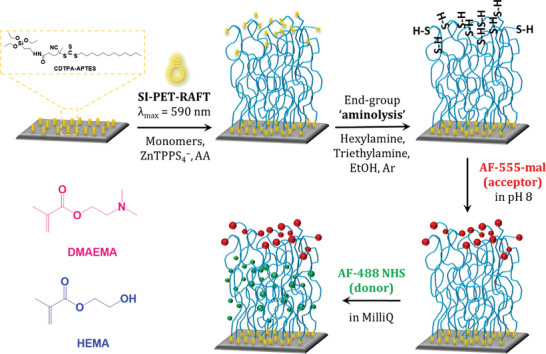
Schematic of SI‐PET‐RAFT polymerization for preparing polymer brush films on substrates previously modified with RAFT chain transfer agents (CDTPA) with functional monomer, 2‐(dimethylamino) ethyl methacrylate (DMAEMA), and 2‐hydroxyethyl methacrylate (HEMA).

Our strategy for integrating FRET chemistry into PEBs is outlined in **Scheme** **1**. Briefly, a triethoxysilane‐functionalized cyano‐4‐[(dodecylsulfanylthiocarbonyl)sulfanyl]pentanoic acid (CDTPA) derivative was synthesized using a literature protocol (Scheme S1 and Figure S1, Supporting Information),^[^ ^18^ ^]^ and was used as a surface‐tethered RAFT chain transfer agent (CTA) initiator. A thin layer of this RAFT CTA (ca. 1.8 nm) was formed after silane‐coupling to an activated Si‐OH wafer/quartz surface. For monomers, we chose the weak polyelectrolyte (DMAEMA), which becomes non‐charged at a specific pH. These were grown together with 1% (v/v) of hydroxyethyl methacrylate (HEMA). The purpose of HEMA was to provide a functional “handle” for coupling fluorophores post‐polymerization. Previous work by us^[^ ^19^ ^]^ validated this doping method for linear diblock random copolymers, using a HEMA‐based monomer as the dopant into NIPAM. We anticipate that the distribution of the HEMA monomer will be random throughout the p(DMAEMA), given that HEMA and DMAEMA have a very similar reactivity to SI‐PET‐RAFT polymerization with the same CTA, with growth rates of 0.16 and 0.22 nm min^−1^, respectively.^[^ ^18^ ^]^ For synthesis, a pre‐polymerization solution comprising the monomer mixture, an oxygen quencher (ascorbic acid), and the photocatalyst (Zn (II) meso‐tetra(4‐sulfonatophenyl) porphyrin (ZnTPPS^4−^)) was allowed to react under λ_max_ = 590 nm LED light irradiation, with a light intensity of 1.82 mW cm^−2^.^[^ ^20^ ^]^ After polymerization, the brush thickness was quantified by spectroscopic ellipsometry. For integrating FRET fluorophores into the brushes, we opted for a strategy of first removing the CTA end‐groups from the brushes, with subsequent thiol‐maleimide coupling with a FRET acceptor (Alexa‐Fluor 555‐maleimide). Subsequently, a FRET donor (Alexa‐Fluor 488‐NHS) was coupled to HEMA within the brush. This way, the acceptors were on the end‐groups, while the donors were randomly conjugated within the brush. In this approach, the FRET output should reflect chain conformations, as the ensemble average distance between donors and acceptors is modulated by the polymer backbone in response to pH.

**Figure 1:**
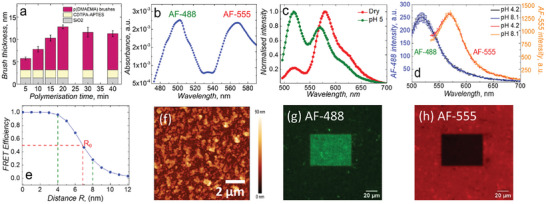
a) Growth kinetics of p(DMAEMA) brushes by spectroscopic ellipsometry, b) UV–vis spectrum of fluorophores conjugated dry brush surface, c,d) emission spectra collected after exciting with *λ*_abs, max_ = 460 nm for c) dry/wet (pH 5) p(DMAEMA) brushes, d) pH‐insensitive AF‐488‐NHS (0.13 µm) and AF‐555‐Maleimide (0.16 µm) after excitation with *λ*_ex_ = 455 nm and 490 nm, respectively, e) calculated dependence of FRET efficiency on an isolated donor and acceptor system as a function of separation distance, and f) dry p(DMAEMA) brushes topography and roughness determination by AFM. The photobleached square region on p(DMAEMA) brushes across the FRET g) donor h) acceptor channels.

A systematic study into the growth kinetics of the PEBs at different time intervals was conducted using spectroscopic ellipsometry where a steady increase in brush thickness was observed, followed by saturation (**Figure** **1**a), which is typical for polymer brushes. This can be explained by the simultaneous formation of high molecular weight polymers in solution distant from the substrate by free radical polymerization which increases solution viscosity and inhibits further polymer brush growth.^[^ ^20^ ^]^ Further FRET modifications were made to p(DMAEMA) brushes with a dry height of roughly 10 nm. These modifications with integrated FRET did not change the overall static water contact angle, *θ*, of ≈53° (Figure S2, Supporting Information)^[^ ^18^ ^]^ similar to the brush without the fluorophores attached, proving that our strategy has not altered the wettability of the surfaces. UV–vis spectra revealed absorption maxima at *λ*_abs, max_ = 501 and 566 nm, corresponding to covalently attached AF‐488 and AF‐555 to the brush surfaces (Figure 1b). Importantly, the UV–vis spectra indicated an approximately equal concentration of both donors and acceptors. With the assumption that all end‐groups were successfully conjugated with AF‐555, we estimate that there were approximately one of each fluorophore per chain, based on our feedstock of 1% v/v of HEMA in the pre‐polymerization solution. We point out that this strategy will likely not hold for every system, as brushes of significantly greater thicknesses would pose challenges regarding the transport of the AF‐488 throughout the brush in a post‐polymerization modification.

Surface fluorescence spectra of p(DMAEMA) were acquired under both dry and wet conditions, utilizing an excitation wavelength of 455 nm (Figure 1c). Brushes in the dry state (collapsed) have the donor and acceptor fluorophores at the closest proximity, so an increased energy transfer among them was observed. In contrast, in the wet state (swollen), a larger ensemble average distance between fluorophores leads to less FRET coupling. AF‐488 and AF‐555 fluorophores (Figure S3, Supporting Information) were purposely chosen and incorporated in brushes as pH‐insensitive amplification motifs (Figure 1d). The distance between the donor and acceptor as well as their relative orientation define the FRET efficiency (E), which was calculated using the formula E = 1/(1+(R/R_0_)^6^),^[^ ^21^ ^]^ where R is the donor‐acceptor distance and R_0_ is the distance at which the FRET efficiency is at 50% (the Forster radius). This calculation can allow the distance dependencies for FRET to be better contextualized. As represented in Figure 1e, FRET efficiency declines as a function of donor‐acceptor distance (R), especially between 4–9 nm with a Forster radius (R_0_) at ≈7 nm. The distance range where the FRET efficiency drops from 0.95 to 0.15 is indicated by green lines, while red lines represent the R_0_. Beyond this distance, there are almost no changes in FRET efficiency. In a simplistic picture, Figure 1e can indicate what the efficiency would be for a single chain if the AF‐488 was conjugated at a distance R from the end‐group (i.e., the end‐group situated at R = 0 nm), suggesting the greatest efficiency if the HEMA monomer is incorporated closer to the chain ends. However, this is too simplistic for the brush system, as there are neighboring interactions to consider over multiple chains in the volume defined by R. Furthermore, the greatest efficiency is not the goal, rather the greatest change in the efficiency as a function of brush conformation (i.e., sensitivity) is desired. The most sensitive architecture for arranging the fluorophores in a brush system is currently the subject of an up‐coming study by us.

The polyelectrolyte brushes were relatively homogeneous, with a surface roughness of 10 nm over an area of 10 × 10 µm (Figure 1f), derived from a root‐mean‐square (RMS) roughness. The acceptor photobleaching method was used to prove the FRET integration using CLSM. The underlying concept is that when the brushes are dry, the donor fluorescence intensity is quenched due to resonance energy transfer to the acceptor, however, once the acceptor is photobleached, de‐quenching in donor intensity (i.e., an increase) is observed. In this study, CLSM measurements of p(DMAEMA) brush surfaces with both the acceptor channel (width between 572 and 703 nm, with λ_ex_ = 543 nm) and donor channel (width between 493 and 548 nm, with λ_ex_ = 488 nm), after the rectangular section was photobleached with λ_ex_ = 543 nm for 50 min at maximum intensity (Figure 1g,h). Since Alexa‐Fluor fluorophores exhibit a high photostability, a longer acceptor photobleaching was needed. As expected, after 50 min, the donor fluorescence was significantly enhanced where the acceptor was bleached, as demonstrated clearly in Figure 1g,h, respectively, confirming the FRET pairing for the brush‐coated surfaces. The fact that the intensity ratio of the donor outside to inside the bleached area matches the ratio of the acceptor inside to outside further ensures that only the acceptor was photobleached and not the donor (Figure S4, Supporting Information).

**Figure 2:**
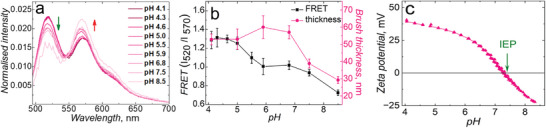
Sensing of local pH. a) Fluorescence emission spectra recorded at a fixed excitation wavelength of *λ*_ex_ = 455 nm, b) overlaying FRET ratio (I_520_/I_570_) along with the brush thickness, and c) zeta potential of p(DMAEMA) brush‐coated surfaces on silicon or quartz substrates as a function of pH using 5 mm of electrolyte solution. pH was adjusted by 5 mm HCl and KOH solutions. In situ ellipsometry measurements were performed at 21 °C. Error bars indicate standard deviations from n = 3 measurements.

To probe the brush FRET as a function of conformation, we chose to use pH as a stimulus, at a constant ionic strength. The effect of solution pH on the average chain conformation for weak p(DMAEMA) PEBs was studied by fluorescence spectroscopy. In an acidic medium, p(DMAEMA) brushes are mostly protonated^[^ ^22^, ^23^ ^]^ and therefore extended due to charge repulsion between monomer units. In this situation, the distances between the donors (AF‐488) and acceptors (AF‐555) is large, therefore energy transfer between them is less feasible (low FRET). However, for brushes in the collapsed state, there is less distance between fluorophores (high FRET). As can be seen from **Figure** **2**a, for FRET pairing in p(DMAEMA) brushes, the donor emission peak at 520 nm wavelength continuously decreased while that of the acceptor at 570 nm increased, in going from the acidic environment to basic, meaning that there was an increase in energy transfer between the two fluorophores as the pH is increased. Moreover, a drastic communal switch in chain conformation above pH 7.0 occurs, closer to the pKa (7.4) of p(DMAEMA).^[^ ^24^ ^]^ Next, the ratio of the FRET donor‐to‐acceptor peak was used to further analyze FRET pairing and was subsequently overlaid with minute variations in wet thickness that were transduced by in situ ellipsometry (Figure 2b; Figure S5 and Table S1, Supporting Information). This is an important parameter for realizing small variations in the chain conformation with that of the FRET ratio.

We represent the FRET (donor‐to‐acceptor) by I_520_/I_570,_ where I_520_ is the intensity of emission at 520 nm, and I_570_ is the intensity of emission at 570 nm, corresponding to the donor and acceptor emission, respectively. In these terms, an increase in I_520_/I_570_ indicates less FRET, while a decrease indicates stronger FRET. Importantly, the start of polymer brush collapse for this system was consistent when compared directly to polymer brush height, demonstrating agreement between FRET and polymer brush height. That is, at a low pH value of 4, p(DMAEMA) brushes were fully charged which was reflected by both the FRET ratio and thickness, confirming a swollen brush, while above pH 7, a drastic decrease in both FRET ratio and brush height was seen, confirming brush collapse. Therefore, typical pH‐mediated transitions were observed (i.e., FRET ratios consistent with collapsed). Interestingly, the modulation of FRET was stronger than that for brush height, indicating an increased sensitivity for our system based on FRET rather than brush height alone. We note that the transitions in brush height with pH are consistent with other studies of similar p(DMAEMA) brush thicknesses,^[^ ^25^ ^]^ indicating that the ellipsometry fitting was not inducing errors in the sensitivity with pH. The greater sensitivity of the FRET may indicate a re‐arrangement of the chains within the brush (i.e., the swelling and collapse that is not purely in the perpendicular direction to the surface) with pH which is not readily reflected in brush height alone, as seen previously for different FRET brush systems.^[^ ^15^ ^]^ The chain rearrangements can be understood in terms of the deprotonation of the p(DMAEMA) with pH, as when the charge is eliminated, the remaining protonated monomers would adapt by finding the greatest separation to neighboring charged monomers, due to electrostatic repulsions. This adaptation would not necessarily affect brush height, at least until a threshold is reached whereby the brush becomes more hydrophobic (charge neutral). Once the pH approaches and surpasses the pKa (charge neutral state), substantial rearrangements in polymer chains takes place, such that the proximity between the donor and acceptor fluorophores is the closest, thereby, increasing the overall FRET.

Conformational changes in PEBs depend highly on their degree of charging. Therefore, streaming zeta potential measurements were conducted, where the effect of solution pH on the surface charges was investigated by injecting 5 mm of electrolyte solutions at different pH (Figure 2c). Figure 2c demonstrates a consistent drop in the zeta potential of p(DMAEMA) brushes, which relates to the deprotonation process. The isoelectric point (IEP), or pH value at which the surface carries zero net charge, is indicated by the intersection of the zeta potential curve of the p(DMAEMA) surface and the horizontal line at zero zeta potential. A further drop in the zeta potential relates to the adsorption of OH^−^ ions on the uncharged brush surface once pH 7.3 was reached (i.e., pKa of the brush surface was reached).^[^ ^26^ ^]^ Importantly, the zeta potential shows changes in the charge even when the brush height is not changing, but where the FRET signal does show changes. This further supports that the FRET method gives increased sensitivity to the chain reorganization with pH.

**Figure 3:**
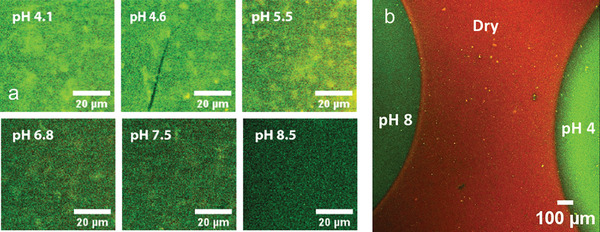
a) CLSM composite channel images of p(DMAEMA) brushes in different pH droplets with the same spectral gating, and b) FRET composite image (green and red channel combined) of two different pH droplets (pH 4 and 8) on a p(DMAEMA) brush surface. Scale bars are 20 and 100 µm, respectively.

A key outcome of integrating FRET chemistry into PEBs is that chain conformations can be spatially and temporally resolved in high resolution using CLSM. **Figure** **3** shows composite CLSM images of the p(DMAEMA) brush surfaces with varying pH, which were used to record these fluorescence changes. A clear distinction in the output of the brushes between acidic and basic pH was visible, where there were clear differences in the green component of the composite images. The FRET increased at basic pH giving lower green color emission, while in acidic pH, a greater green emission was observed (Figure 3a). To illustrate the feasibility of spatially probing the chain arrangement on FRET‐integrated brushes, two droplets of different pH, i.e., pH 4 and pH 8, were brought into close proximity (Figure 3b). These first results present an opportunity to “see and resolve” conformational changes toward the nanoscale in a complex microenvironment. Interestingly, we could identify a halo around the bad solvent droplet (pH 8), which indicated some swelling, but different from the bulk droplet. This may be indicative of some ionic or pH gradients as the brush is solvated with air above.

**Figure 4:**
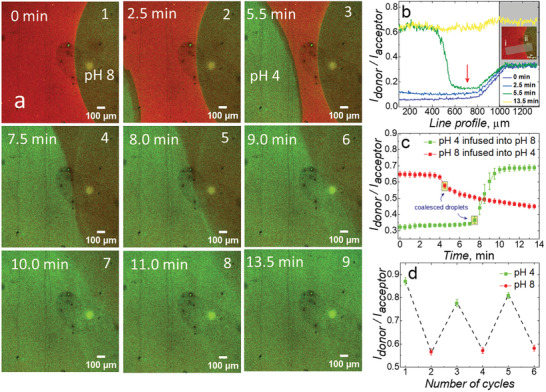
a) FRET‐composite confocal images b) normalized line profiles analysis for images 1, 2, 3, and 9 of a), c) swelling and collapse kinetics across donor and acceptor channels for a p(DMAEMA) brush surfaces in 2.5 µL pH 8 droplet infused with pH 4 solution as a function of time with 5 µL min^−1^ flow rate d) FRET transition cycled between pH 4 and pH 8 droplets. Light grey box in b) indicates the approximate region over which the line profile was calculated (shown in inset). The CLSM composite measurements had the acceptor channel set with width between 572 nm and 703 nm, and the donor channel with width between 493 and 548 nm, both channels excited by λ_ex_ = 488 nm.

To elucidate the real‐time switching properties of the brushes in acidic (pH 4) and basic (pH 8) solutions, CLSM was utilized. Initially, a microdroplet of pH 8 solution was placed on a brush, with an aqueous pH 4 solution infusing slowly toward the initial droplet via a syringe pump at a speed of 5 µL min^−1^. Any changes in intensities of the donor and acceptor channel were monitored as a function of time upon exciting the donor. The initial droplet was watched via bright field imaging until it stopped advancing (after ≈10 s). CLSM images were taken every 30 s until the second microdroplet coalesced and fully flushed away with the acidic aqueous solution (images 1 to 6 in **Figure** **4**a). For the initial droplet, at 0 min before infusing pH 4 solution, a drastic difference between the wet (bad solvent, green color droplet referring to a nearly collapsed state) and dry phase (red color, fully collapsed chains) was clear, along with the typical hydration ring outside of the contact line. When the second droplet was approaching (images 2–3), interestingly, the brush was changing its solvated state in the place between the two droplets, likely occurring from the humidity surrounding the second droplet. Furthermore, once the droplets had fully coalesced (from image 4), there was a clear memory of the initial bad solvent droplet that persisted, eventually recovering toward the final image (image 9), demonstrating a delay in the brush responses when there is mixing solvent from above. Previously, memory effects have been observed in PNIPAM brushes under co‐nonsolvency conditions,^[^ ^27^, ^28^ ^]^ which result from molecular reorientations of the brush in response to the solvent, which persists afterward (i.e., a memory of the solvation installed into the brush). These memory effects in the FRET brush likely represents a similar phenomenon. The delay in equilibrium in the brush once the droplets coalesced, with a visible memory of the contact line area (images 4 to 8) cannot readily be probed in such resolution by other methods, to our best knowledge. These transitions were further analyzed by extracting the line profiles of the composite channel where the ratio of donor to acceptor intensities was considered (Figure 4b) for select images (for ease of comprehension) and Figure S6 (Supporting Information) (for all images). What can be interpreted from the line profile is that the dry state (left side) had a higher FRET than the pH 8.0 droplet (right side), and as the pH 4.0 droplet approaches (from left side), a steady decrease in FRET was observed, consistent with a “swelling” of the brush between the two droplets (red arrow in Figure 4b). Time after the droplets coalesced, the FRET was consistent with that of the pH 4.0 droplet, due to an excess of the pH 4.0 solution being injected continuously across the field of view. The reverse system (pH 8.0 droplet into pH 4.0 droplet) was also studied (Figure S7a,b, Supporting Information), which showed similar, albeit reverse behavior.

To better understand the conformational kinetics, the FRET of the initial droplet region of Figure 4b (far right) was monitored with time (Figure 4c). Note that differences in FRET between CLSM and fluorescence are attributed to the inherent differences in the experimental parameters, such as the spectral gating in CLSM, and therefore should not be directly compared to the fluorescence spectra. From Figure 4c, the pH 8 droplet, within 2 min of coalescing with the pH 4 droplet (green curve), the swelling transition was fast with a plateau after about 2 min (Video S1, Supporting Information). However, for the reverse system (pH 8.0 droplet coalesced with pH 4.0 droplet), the transition was significantly slower, where the FRET was still changing after 10 min (Video S2, Supporting Information). This signifies that the collapsed‐to‐swollen transition occurs considerably more quickly. Interestingly, our results are consistent with some previous works^[^ ^29^, ^30^ ^]^ where kinetics studies on p(DMAEMA) brush surface with QCM‐D and in situ ellipsometry were performed. Mechanistically, during the swelling process, protonation of the outer brush chains happens faster allowing for further uptake of solvent and counterions until all monomer segments are fully protonated,^[^ ^31^ ^]^ whilst brush collapse is a slower process due to deprotonation starting from outer brush chains bringing pH gradients into the brush. These findings suggest that, in contrast to the rapid kinetics of protonation and chain disentanglement seen in the p(DMAEMA) brush swelling transition, the release of protons and solvent from the collapsing brush can occur gradually. Additionally, Figure 4d illustrates the switching of the FRET response with different pH droplets, cycled three times between pH 4 and 8 (CSLM images, Figure S8, Supporting Information). Our method provides strong support to the fundamental investigations on the solvation of PEBs, their response to solvation, and their capability to resolve temporal changes in complex microenvironments toward the nanoscale. This heightened sensitivity could provide new perspectives on physicochemical processes occurring at these interfaces.

Taken together, this new strategy for integrating FRET chemistry into PEBs can offer a new basis for probing complex changes in polymer brush dynamics that are otherwise inaccessible by traditional macroscopically limited methods.^[^ ^13^ ^]^ Moving forward, we anticipate an exciting extension of this work by coupling brush chain transitions to fluorescence lifetime imaging microscopy (FLIM), where FLIM can provide further sensitivity by revealing the intensity and lifetime of the FRET output.^[^ ^32^ ^]^ Furthermore, our new approach for integrating FRET chemistry into polymer brushes can now be used as a basis for developing other sensing materials that leverage other functional polymer brushes.

## 3 Conclusion

Polyelectrolyte brushes with specific fluorophore architectures were prepared using SI‐PET‐RAFT polymerization, with post‐modification with capabilities for FRET‐based conformational signaling. These were developed to spatially resolve polyelectrolyte brush conformational changes upon different solvation processes (e.g., swelling, collapse, memory effects). These brushes were shown to produce FRET modulation as a function of stimuli that affects polyelectrolyte conformation (herein chosen as pH at constant ionic strength), reversibly, and in real‐time. This allowed for probing conformational transitions of chains by CLSM with high spatial (hundreds of nm) and temporal (sub‐seconds) resolution. This resolution is far greater than other methods for resolving polyelectrolyte brush conformational changes, which tend to be macroscopically limited (10 s of micrometers) with lengthy acquisition times (10 s of minutes). These surfaces were used as a basis for sensing polymer conformation around complex interfaces of droplets containing different stimuli (of different pH). We observed interesting swelling effects of polyelectrolyte brushes by droplets, memory effects of chain conformation, and interesting mixing dynamics as stimuli were switched, which are not readily possible in such resolution by other methods, to our best knowledge. Our surface‐based sensing platform gives a greater spatial resolution that has not been achieved previously, to our best knowledge. It is anticipated that these conformational sensing surfaces will have broad applications for probing subtle changes in chemical and biological systems.

## 4 Experimental Section

### Materials

All chemicals were of analytical grade and used as received without purification, except for 2‐hydroxyethyl methacrylate (HEMA) and 2‐(dimethylamino)ethyl methacrylate, DMAEMA, which were purified by passing through a short aluminium oxide column before reaction. High‐purity water (MilliQ water) with a resistivity of >18.2 MΩ cm was obtained from an inline Millipore RiOs/Origin water purification system (Millipore Corporation, Massachusetts, USA). Polished single‐crystal (100)‐silicon wafers were obtained from Silicon Materials, Kaufering, Germany, with a native SiO_2_ layer thickness of ≈1.5 nm. Optical fused quartz square coverslips (22 × 22 × 0.2 mm) were obtained from Micro to Nano (Haarlem, The Netherlands). Compounds DMAEMA, HEMA, 1‐Ethyl‐3‐(3‐dimethylaminopropyl)carbodiimide hydrochloride (EDC), 3‐Aminopropyltriethoxysilane (APTES), cyano‐4‐[(dodecylsulfanylthiocarbonyl)sulfanyl]pentanoic acid (CDTPA) hexylamine, ethylamine, dichloromethane (DCM), hexane, ethanol, and 1‐propanol were obtained from Sigma‐Aldrich. Zn(II) meso‐tetra(4‐sulfonatophenyl)‐porphyrin (ZnTPPS^4−^) was obtained from Frontier Scientific and used as received. Thorlabs Olympus BX & IX series (λ_max_ = 590 nm) collimated light‐emitting diodes (LEDs) were used for all light‐mediated reactions. LED light irradiance was modulated by a Thorlabs LED DC4100 4‐channel LED driver. The irradiance was measured using a Thorlabs PM100D‐Compact Power and Energy Meter Console. CDCl_3_ was obtained from Eurisotop (Saint‐Aubin, France).

### Synthesis of CDTPA‐APTES RAFT Agent

A 250 mL flask equipped with a magnetic stir bar and rubber septum was charged with cyano‐4‐[(dodecylsulfanylthiocarbonyl)sulfanyl]pentanoic acid (CDTPA) (403.6 mg, 1 mmol) and DCM (40 mL). N‐(3‐dimethylaminopropyl)‐N′‐ethylcarbodiimide hydrochloride (EDC HCl) (191.7 mg, 1 mmol) dissolved in DCM (10 mL) was then added dropwise into the flask. The solution was then cooled and stirred at 0 °C for 10 min. Sequentially, (3‐aminopropyl) triethoxysilane (APTES) (0.23 mL, 1 mmol) was added dropwise into the solution. The reaction mixture was stirred at 0 °C for 2 h, then at room temperature for 2 h, and then concentrated in vacuo. The crude product was purified with silica gel column chromatography (2:3 v/v ethyl acetate and hexanes) to provide CDTPA‐APTES as a viscous yellow liquid.^[^ ^20^ ^] 1^H NMR (500 MHz, CDCl_3_, 30 °C, δ, ppm): 0.63 (s, 2H), 0.88 (t, 3H), 1.26 (t, 9H), 1.38 (m, 16H), 1.66 (m, 6H), 1.91 (s, 3H), 2.45 (m, 4H), 3.29 (t, 4H), 3.83 (q, 6H), 5.88 (t, 1H) (Figure S1, Supporting Information).

### Synthesis of p(DMEAMA‐HEMA) Copolymer Brush Surfaces Using SI‐PET‐RAFT

Silicon wafers (20 × 13 mm) or fused quartz coverslips (22 × 22 × 0.2 mm) were treated with EtOH in an ultrasonic bath for 20 min at 37 °C and then dried under nitrogen. The cleaned substrates were then activated in an oxygen plasma for 1 min (Harrick, Plasma Cleaner PDC‐002 with Plasma Flo PDC‐FMG) After cleaning, the wafers were placed in a dilute solution containing 20 µL of synthesized CDTPA RAFT in 35 mL of dry toluene, overnight at room temperature. After 24 h, the CTA‐functionalized substrates were rinsed thoroughly with toluene, and isopropanol, and dried under a stream of nitrogen gas. These substrates were then subjected to polymerization. Unless otherwise noted, all reactions were placed ≈13 cm below an LED light source (adjusted to 1.82 mW cm^−2^ intensity). A stock solution containing 0.5 mg of photocatalyst (ZnTPPS^4−^) in 0.5 mL MilliQ was prepared in a vial and stored in the dark. Meanwhile, HEMA and DMAEMA were purified through a basic alumina column to remove any inhibitor. The inhibitor‐free monomers (DMAEMA or HEMA), ascorbic acid (AA), and the ZnTPPS_4_^−^/MilliQ pre‐polymerization stock solution mixed with a molar ratio of [monomer]:[ascorbic acid]:[ZnTPPS_4_^−^] = **500:2:0.025** was freshly prepared every time.

### Preparation of p(DMAEMA‐HEMA) Copolymer Brushes

A CTA‐functionalized native oxide silicon wafer substrate was placed on top of a glass base plate. Then, 150 µl from the reaction mixture containing DMAEMA (1.21 ml), and HEMA (9.3 µl) with AA (5.4 mg) in 0.5 ml ZnTPPS_4_^−^/MilliQ stock were dropped onto the substrate. A glass coverslip was placed on top of the substrate to form a thin layer of solution. Unless otherwise noted, each surface was irradiated with λ_max_ = 590 nm light at an irradiance of I = 1.82 mW cm^−2^ for 30 min. After irradiation, the substrates were thoroughly rinsed with DI water followed by isopropyl alcohol and then dried under a nitrogen stream.

### Conjugating AF‐555 maleimide (Acceptor) and AF‐488 NHS (Donor) to Polymer Chains

Conjugation of AF‐555 maleimide on polymer chains was done with thiols after removing the chain‐end CTA group. For that, first, 5 mL ethanol with hexylamine (10 µL, 0.765 mmol), and triethylamine (10 µL, 0.715 mmol) in equimolar amounts were mixed and argonated for ≈15 min. This mixture (500 µL) was added to each brush substrate and left for an hour. It was later rinsed thoroughly with ethanol and dried with Ar. Each surface was covered with a solution of 0.5 µL AF‐555 mal in 500 µL KCl electrolyte solution of pH 8. This modification was allowed for ≈2 h following rinsing and drying with MilliQ. Utilizing 0.5 µL of AF‐488 NHS in 500 µL of MilliQ, the final modification was carried out on the hydroxyl groups of HEMA segments. Once again, the surfaces were washed with MilliQ water followed by drying.

### Preparation of pH Solutions

Solutions with different pH values but the same ionic strength were prepared using 5 mM HCl, 5 mm KOH, and 5 mm KCl for investigation of the pH‐responsive properties of PEBs. The solutions of different pH were prepared by adding a small amount of 5 mm HCl or 5 mM KOH to 5 m KCl to obtain target pH values with the same ionic strength. Buffered pH solutions were not used here to avoid the generation of specific ion effects.^[^ ^33^ ^]^

### Fluorescence and UV–vis Measurements

All measurements were performed with a multimode microplate reader (Tecan Spark 10 m, Switzerland) using 6‐well glass bottom fluorescence plates with a thickness (0.17 ± 0.005 mm from Cellvis). The UV–vis spectra of all the modifications were performed. Fluorescence spectra were recorded (λ_ex_ = 455 nm, dλ = 2 nm, 460 – 700 nm) whilst maintaining the temperature at 24 °C.

### Water Contact Angle (WCA)

Contact angles of freshly purified water (conductivity of 0.055 µS cm^−1^) from the MicroPure UV/UF device (Thermo Electron LED GmbH) were measured with OCA35XL from Data Physics. Recorded contact angles were analyzed using the SCA20 program from Data Physics. The measurements were performed at a temperature (22.5 ± 0.5%) in a temperature‐controlled lab.

### Confocal Laser Scanning Microscopy (CLSM)

A combined setup of an Axio Observer Z.1 inverted microscope with an LSM710 confocal laser scanning module (Carl Zeiss Microscopy, Germany) was used for spatially‐resolved fluorescence studies. 10x objectives were used. The donor was excited by an argon laser (488 nm with a laser intensity of 2) and the acceptor with a helium‐neon laser (543 nm with a laser intensity of 2). The pinhole and gain were set to 1 AU and 800, respectively. Note that for the acceptor photobleaching experiment, 100 percent intensity of 543 nm laser was used.

### Atomic Force Microscopy (AFM)

AFM measurement were performed in ambient conditions with PeakForce Tapping mode on a Dimension FastScan AFM (Bruker Nano Surfaces and Metrology, CA, USA) with a Scanasyt Fluid+ cantilever (Bruker AFM Probes Nanofabrication Center, Ca, USA) with a nominal spring constant of 0.7 N/m and tip radius of 2 nm. The measurement was performed with a frequency of 8 kHz, amplitude of 100 nm, and a scan rate of 2 Hz at 512 px/lines.

### Nuclear Magnetic Resonance (NMR) Spectroscopy

The ^1^H (500.13 MHz) NMR spectra were recorded using an AVANCE III 500 Spectrometer (Bruker, Germany) using CDCl_3_ at 30 °C.

### Ellipsometry

The ellipsometry experiments were conducted at a constant temperature of 21 ± 0.1 °C, with data collected at an incidence angle of 70° in the range of 600–850 nm to exclude interference from fluorophore absorption. A three‐layer optical model consisting of Si/SiO_2_/Cauchy layer with atmosphere compensation in the VASE software was used to fit the experimental data. The first layer is a substrate layer of Si, the second layer is a native oxide layer with a thickness of 1.5 nm, and the third layer is a Cauchy model (n = A+B/λ^2^, k = 0), which corresponds to the optical properties of the p(DMAEMA) brush. In this model, A and B are fit parameters, and n and k are the real and imaginary components of the index of the refraction, respectively. An isotropic refractive index of n = 1.439 for p(DMAEMA) was fixed for dry polymer brushes. However, for conducting in situ real‐time experiments, the solvated brush thickness, angle offset, as well as A and B parameters were fitted since the brush thickness was above 10 nm. The average value of at least three measurements on different locations for each surface was reported.

### Streaming Surface Zeta Potentials Measurements

An electrokinetic analyzer (SurPass, Anton Paar) comprising an adjustable gap cell was used to determine the zeta (ζ) potential of the planar surfaces. Two sample surfaces with a rectangular size of 1 cm × 2 cm were accommodated into the cell and placed parallel to each other, forming a micro slit with a separation of 100 µm. The measurements were conducted by forcing 5 mm KCl electrolyte solution through the channel via syringe pumps. The pH of the electrolyte was adjusted from 4 to 8 stepwise with auto pH titration by adding diluted KOH. ζ potential was determined from streaming current data using the Helmholtz–Smoluchowski equation and the Fairbrother–Mastin approach.

## Conflict of Interest

The authors declare no conflict of interest.

## Supporting information



Supporting Information

Supplemental Video 1

Supplemental Video 2

## Data Availability

The data that support the findings of this study are available from the corresponding author upon reasonable request.
