# Supplementary material for: Capturing Conformational Transitions of Fluorescently‐Coupled Polyelectrolyte Brushes with High Spatiotemporal Resolution
Source: Small. 2025 Jan 21;21(8):2409323. doi: 10.1002/smll.202409323 (PMC11855223; doi:10.1002/smll.202409323)
Supplement: Supplementary file 1 — Supporting Information [file SMLL-21-2409323-s003.docx]

Supporting Information

Capturing Conformational Transitions of Fluorescently-Coupled Polyelectrolyte Brushes with High Spatiotemporal Resolution

Jyoti Yadav*, Ilka Hermes, Andreas Fery, Quinn A. Besford*

Scheme S1. Synthetic modification of CDTPA with APTES.


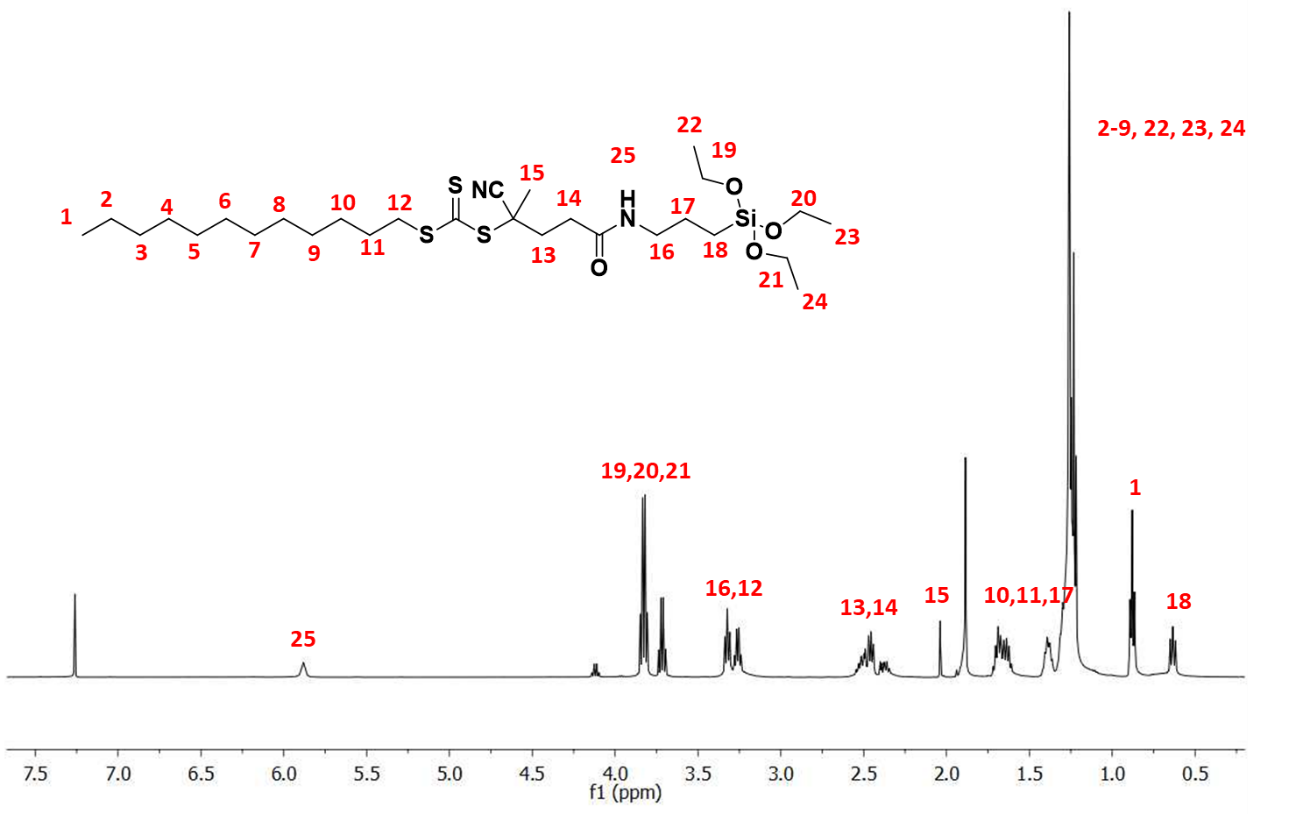


Figure S1. ^1^H NMR spectra of CDTPA-APTES in CDCl_3._

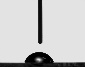

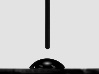

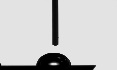

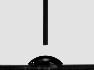

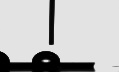


Figure S2. Water contact angle (WCA, *θ*) of sequentially modified p(DMAEMA) brush substrates.

Figure S3. Molecular structures of pH-insensitive Alexa fluor 488-NHS (donor) and Alexa fluor-555 maleimide (acceptor).

Figure S4. Normalised line profile across the acceptor photobleached area on p(DMAEMA) brush surface.


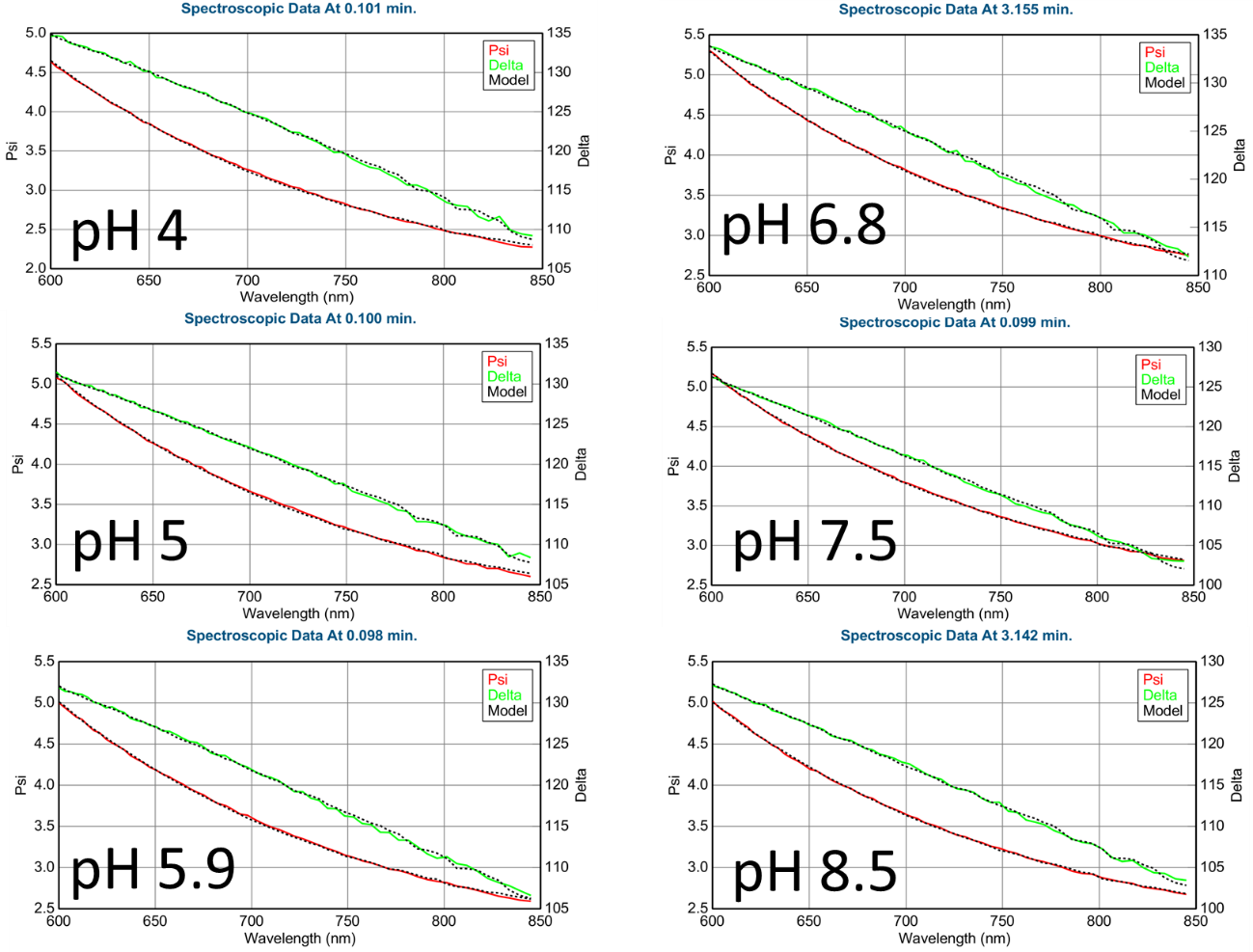


Figure S5. Spectroscopic in-situ ellipsometry data and modelled fits for deriving the brush heights as a function of pH.

Table S1. (a) Example of a three-layer optical model consisting of a silicon substrate layer at the bottom, followed by a 1.5-nm-thick native silicon oxide layer, and then a pDMAEMA brush layer was used. (b) Example thicknesses and optical constants of polymer film layers that were fitted for each pH studied, along with MSE values of the fits, and corresponding example heights, given with standard errors.


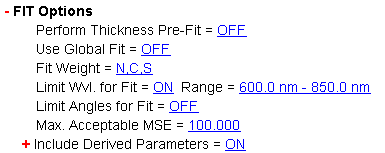

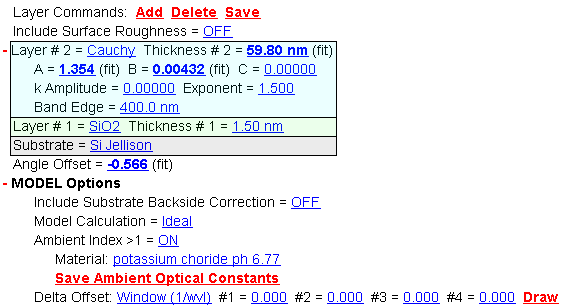


(a)

(b)

| pH | A | B | MSE | Height |
| --- | --- | --- | --- | --- |
| 4.0 | 1.354 ± 0.00091 | 0.00449 ± 0.000091 | 0.444 | 49.18 ± 1.32 nm |
| 5.0 | 1.361 ± 0.00099 | 0.00490 ± 0.00011 | 0.454 | 47.41 ± 1.12 nm |
| 5.9 | 1.355 ± 0.00075 | 0.00411 ± 0.000091 | 0.469 | 56.74 ± 1.20 nm |
| 6.8 | 1.354 ± 0.00059 | 0.00432 ± 0.00007 | 0.420 | 59.80 ± 1.02 nm |
| 7.5 | 1.378 ± 0.0012 | 0.0040 ± 0.00010 | 0.355 | 41.18 ± 0.78 nm |
| 8.5 | 1.383 ± 0.0018 | 0.00468 ± 0.00012 | 0.364 | 33.80 ± 0.88 nm |

Figure S6. Normalised line profiles of Figure 4a of the main text.


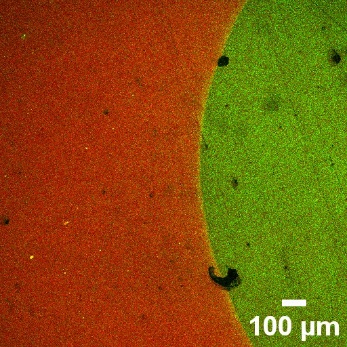

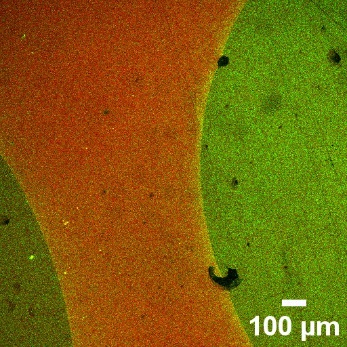

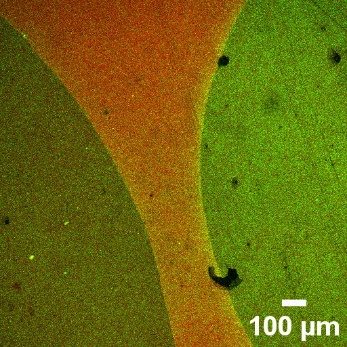

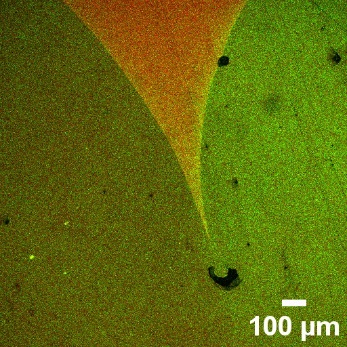

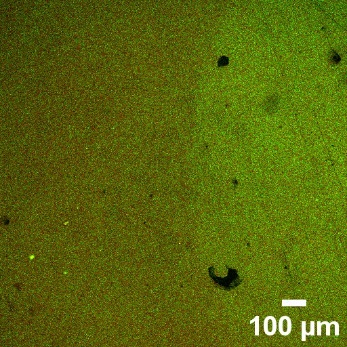

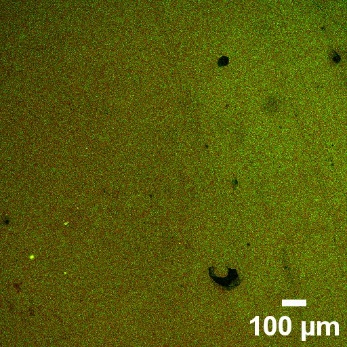

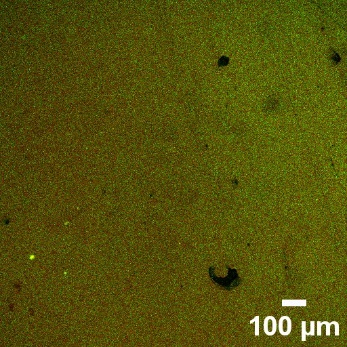

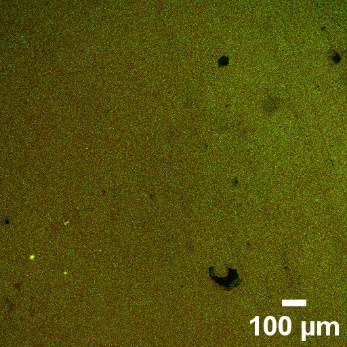


0 min

2.5 min

1

2

5

3

6

4

7

8

3.5 min

4.0 min

4.5 min

7.5 min

13.0 min

13.5 min

a


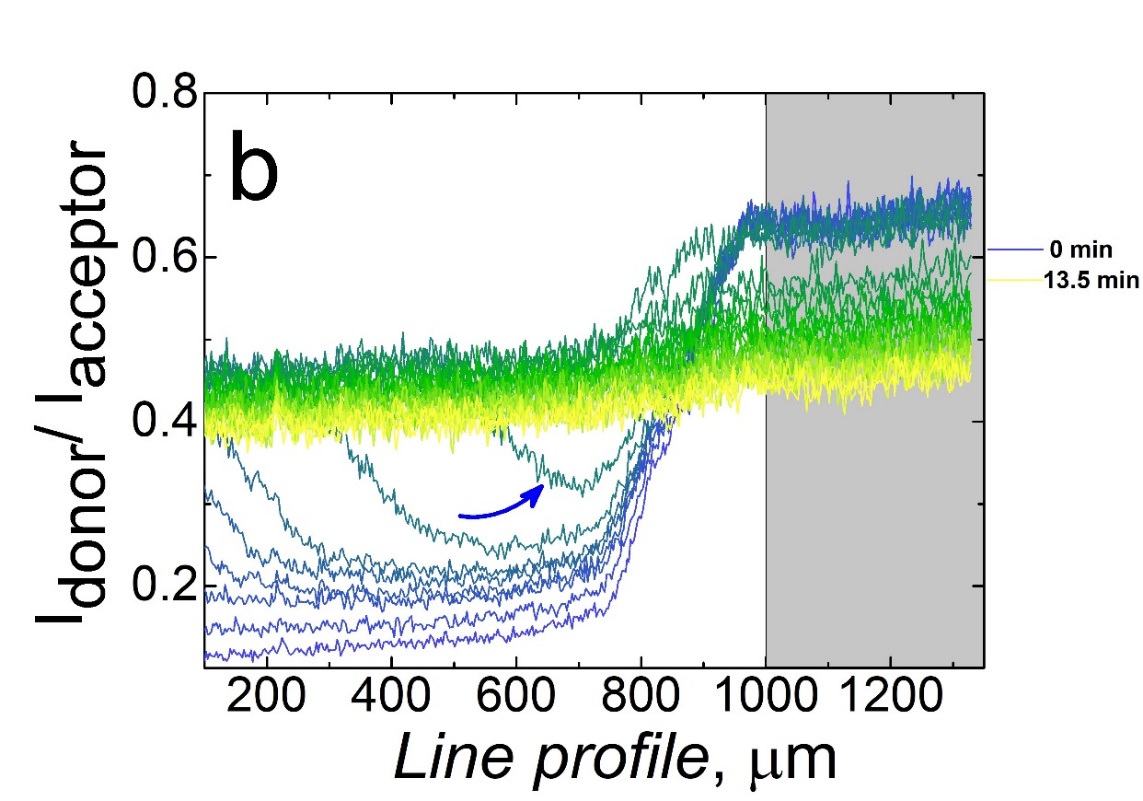

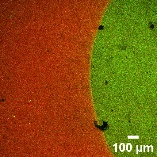


ii

i

Figure S7. (a) CLSM-composite images (b) normalised line profiles of a 2.5 μL pH 4 droplet infused with pH 8 solution on the polymer brush surface as a function of time at 5 µL min^-1^ flow rate.


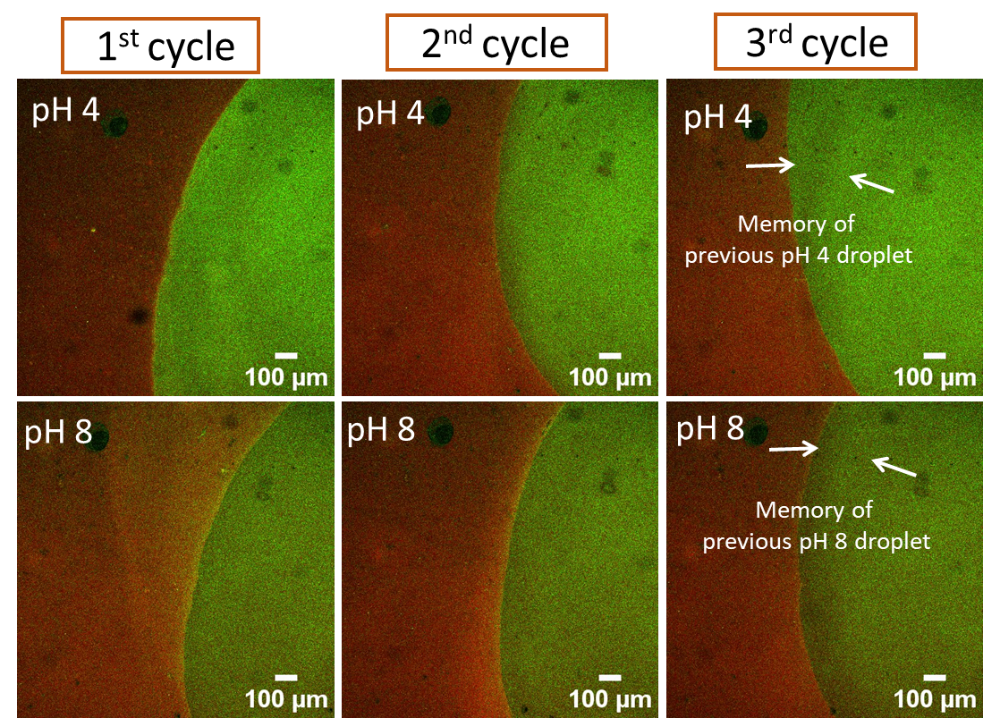


Figure S8. CLSM-composite images of reversible switching in p(DMAEMA) brush surface between acidic and basic pH.
